# A multifunctional oxidative stress nanoamplifier with ROS amplification and GSH exhaustion for enhanced chemodynamic therapy

**DOI:** 10.3389/fphar.2022.1044083

**Published:** 2022-11-10

**Authors:** Wenzhao Zhong, Feng Guo, Fangman Chen, Man-Kay Law, Jun Lu, Dan Shao, Hua Yu, Ging Chan, Meiwan Chen

**Affiliations:** ^1^ State Key Laboratory of Quality Research in Chinese Medicine, Institute of Chinese Medical Sciences, University of Macau, Macau, Macau SAR, China; ^2^ School of Biomedical Sciences and Engineering, South China University of Technology, Guangzhou International Campus, Guangzhou, Guangdong, China; ^3^ State Key Laboratory of Analog and Mixed-Signal VLSI, IME and FST-ECE, University of Macau, Macau, Macau SAR, China; ^4^ State Key Laboratory of Southwestern Chinese Medicine Resources, School of Pharmacy, Chengdu University of Traditional Chinese Medicine, Chengdu, China

**Keywords:** chemodynamic therapy, artesunate, ROS amplification, GSH exhaustion, intratumoral catalytic nanomaterials

## Abstract

Chemodynamic therapy (CDT) eradicates tumors by intratumoral catalytic chemical reaction and subsequently disrupts redox homeostasis, which shows tumor specific reactive oxygen species (ROS)-mediated therapy. However, insufficient ROS generation and high levels of glutathione (GSH) in cancer cells have limited the therapeutic efficacy of CDT. Herein, we constructed a multifunctional oxidative stress nanoamplifier with ROS amplification and GSH exhaustion for enhanced CDT. Such a sandwich-like nanoamplifier comprised layer-by-layer artesunate (AS) and calcium carbonate coatings on the surface of manganese dioxide (MnO_2_) nanoparticles. The nanoamplifier was disassembled under an acidic environment once accumulated into tumor sites, and subsequently released AS to replenish the intratumoral peroxide pool for ROS amplification. Besides being an AS carrier, MnO_2_ exhausted GSH to yield Mn^2+^ ions that catalyzed the overexpression of H_2_O_2_ in the tumor, further intensifying the oxidative stress and facilitating cancer cell death. Taken together, our findings not only provide a paradigm for fabricating intratumoral catalytic nanomaterials, but also present a new ROS enhancement strategy to improve anti-tumor efficacy. Our multifunctional oxidative stress nanoamplifier might broaden the future of CDT.

## 1 Introduction

Dynamic therapy, a clinically promising treatment modality that utilizes *in situ* generation of reactive oxygen species (ROS) to eradicate malignant cancer, has attracted significant attention for its advantages in overcoming multidrug resistant (MDR) cancer (Wang S. et al., 2021). Chemodynamic therapy (CDT) catalyzes intratumoral overexpressed hydrogen peroxide (H_2_O_2_) to generate highly cytotoxic hydroxyl radical (·OH), which circumvents the major limitations of hypoxia and light penetration in clinically approved dynamic therapy including photodynamic and sonodynamic therapy (Tang et al., 2019; Zhou et al., 2021). Although intracellular H_2_O_2_ levels in tumor tissues are higher than normal cells, the endogenous concentration of the peroxide pool is still insufficient for satisfactory chemodynamic efficacy (Zhou et al., 2021). Moreover, overexpressed glutathione (GSH) is the major ROS scavenger in tumor, which plays an important role in protecting cells from various injuries, and increases resistance to chemotherapy, radiotherapy and dynamic therapy (Shao et al., 2020; Chen et al., 2022). Conceivably, GSH is another challenging obstacle for effective CDT in cancer cells, which leads to insufficient radical generation for tumor oxidative damage (Chen et al., 2020; Wang et al., 2020; Shi et al., 2021). Thus, it is imperative but challenging to develop novel Fenton-like mediated ROS nanoamplifiers for GSH exhaustion and peroxide replenishment to enhance the efficacy of CDT.

Manganese dioxide (MnO_2_) can consume intratumoral protons to release Fenton-like Mn^2+^ ions (Wang et al., 2022). In addition, MnO_2_ can directly undergo a redox reaction with GSH to yield glutathione disulfide (GSSG) and Mn^2+^ ions, which enhances CDT by simultaneously generating ·OH and disrupting the cellular antioxidant defense system (Lin L.-S. et al., 2018). It is worth noting that the bicarbonate (HCO_3_^−^) is indispensable for Mn^2+^-mediated Fenton-like reaction (Li et al., 2016). Fortunately, HCO_3_^−^ is one of the important physiological buffers and can be continuously generated via carbonic anhydrase IX (CA IX) for Mn^2+^-mediated Fenton-like reaction to intensify the intratumoral oxidative stress (Stadtman et al., 1990; Yim et al., 1990; Lin L. S. et al., 2018). However, the therapeutic efficacy of Mn^2+^-mediated CDT is also compromised by insufficient H_2_O_2_ (∼100 μM) in tumor microenvironment (Fu et al., 2021; Liu B. et al., 2022). Therefore, it is imperative to develop a peroxide replenishment approach to increase the concentration of intratumoral peroxide species, which overcomes the endogenous H_2_O_2_ insufficiency and achieves satisfactory Mn^2+^-mediated CDT efficacy.

In this regard, there are two main strategies to replenish intratumoral peroxide species including amplification of H_2_O_2_ production and delivery of other peroxide compounds. Some of the natural endoperoxide compounds such as artesunate (AS) can serve as exogenous peroxide species for replenishing the shortage of intracellular peroxide pools and boosting the Mn^2+^-mediated CDT (Lin et al., 2020; Wang D. et al., 2021; Jin et al., 2021). Therefore, delivery of AS demonstrates the prominent advantages in overcoming insufficient H_2_O_2_ for effective CDT. However, administered AS inevitably undergoes rapid decomposition before accumulation in tumor to initiate Fenton-type reactions, resulting in low availability of exogenous peroxide replenishment for ROS production. A nanocarrier with tumor microenvironment-responsive property is thus necessary for controllable release of AS. Recently, calcium carbonate (CaCO_3_) nanomaterials have been utilized as surface coating materials to protect the inner core from leakage or degradation, and to endow controllable degradation with pH-responsive properties for precise cancer therapy (Wan et al., 2019; Chang et al., 2020). The CaCO_3_ layers will first degrade into HCO_3_^−^ and Ca^2+^ ions in acidic tumor microenvironment. The release of Ca^2+^ ions is accumulated within cancer cells, resulting in Ca^2+^ ions overload due to the disturbance of intracellular Ca^2+^ ions homeostasis, which may aggravate the intratumoral oxidative stress (Bagur and Hajnóczky, 2017). Besides, the release of HCO_3_^−^ facilitates the Mn^2+^-mediated Fenton reaction for enhanced CDT. Collectively, the rational integration of AS, CaCO_3_ and MnO_2_ will not only improve the bioavailability and stability of AS, but also amplify the intratumoral oxidative stress through peroxide species replenishment and HCO_3_^−^ ion-assisted CDT based on Mn^2+^-mediated Fenton-like reaction.

Herein, we designed a facile strategy to fabricate a tumor microenvironment-activatable oxidative stress nanoamplifier for enhanced CDT through ROS amplification and GSH exhaustion. The nanoamplifier exhibited a sandwich structure, and the middle layer of CaCO_3_ was coated on the surface of MnO_2_ (MnO_2_@CaCO_3_@AS) to avoid the contact with AS, preventing from premature decomposition of AS. In the design, CaCO_3_ also provides anchor points for AS self-assembly on its surface through the coordination interaction between Ca^2+^ and carboxyl group of AS, achieving high drug loading. The nanoamplifiers were disassembled under mild acidic environment, resulting in the release of AS for peroxide species replenishment. Meanwhile, the exposure of MnO_2_ further consumed GSH to yield Mn^2+^ ions for ROS generation through *in situ* catalyzation of overexpressed H_2_O_2_ and the delivered AS. The PEGylated liposome was coated on the surface of nanoamplifier (MnO_2_@CaCO_3_@AS@PEG, denoted as MCAP) to improve colloidal stability, which facilitated the cellular uptake. Taken together, the MCAP nanoamplifier was designed to amplify intracellular ROS levels and consume GSH, collectively disrupting the intratumoral redox balance to achieve efficient tumor eradication. Our multifunctional oxidative stress nanoamplifiers might provide a facile but intriguing strategy to enhance CDT through peroxide species replenishment, ROS amplification and GSH exhaustion.

## 2 Materials and methods

### 2.1 Chemicals and reagents

Anhydrous calcium chloride (CaCl_2_), poly(allylamine hydrochloride) (PAH, MW∼15,000), poly(acrylic acid) (PAA, MW∼2000), 3,3′,5,5′-tetramethylbenzidine (TMB), methylene blue (MB), amiloride hydrochloride and rhodamine 123 (Rhd 123) were purchased from Macklin Biochemical Co., Ltd (Shanghai, China). Artesunate (AS), glutathione (GSH), sodium bicarbonate (NaHCO_3_), 1-ethyl-3-(3-dimethylaminopropyl)carbodiimide (EDC), cholesterol, chlorpromazine hydrochloride, 5,5′-dithio-bis-(2-nitrobenzoic acid) (DTNB), genistein, colchicine and DMSO were obtained from Aladdin (Shanghai, China). Ammonium bicarbonate (NH_4_HCO_3_) and 3-[4,5-dimethylthiazol-2-yl]-2,5 diphenyl tetrazolium bromide (MTT) were purchased from Sigma-Aldrich (Saint Louis, United States). 1,2-dioleoyl-sn-glycero-3-phosphate (sodium salt) (DOPA) was purchased from Avanti (Alabama, United States). 1,2-dihexadecanoly-sn-glycero-3-phosphocholine (DPPC) was purchased from Xi’an ruixi Biological Technology Co., Ltd (Xi’ an, China). N-(Carbonyl-methoxypolyethylene glycol 2000)-1,2-distearoyl-sn-glyverol-3-phosphoethanolamine sodium salt (DSPE-mPEG_2k_) was purchased from AVT (Shanghai, China). mPEG_2k_-NH_2_ was purchased from ShangHai ToYangBio Tech.Inc. (Shanghai, China). Sodium hydroxide (NaOH) and hydrogen peroxide (H_2_O_2_) were purchased from Xilong Scientific Co., Ltd (Shantou, China). Anhydrous manganese (II) chloride (MnCl_2_) was obtained from Energy Chemical (Shanghai, China). 1,1′-Dioctadecyl-3,3,3′,3′-tetramethylindotricarbocyanine iodide (DiR) was obtained from AAT Bioquest (California, United States). Anhydrous ethanol was obtained from Sinopharm Chemical Reagent., Ltd (Beijing, China). Chloroform was obtained from DAMAO CHEMICAL REAGENT FACTORY (Tianjin, China). Fluo-4 AM, DCFH-DA, Hoechst 33,342 and crystal violet were obtained from Beyotime Biotechnology (Shanghai, China). BODIPY™ 493/503 and ThiolTracker™ Violet were obtained from Thermofisher (Massachusetts, United States). Ultrapure water was obtained from Milli-Q systems (Merck Millipore, United States).

### 2.2 Preparation and surface modification of nanoparticles

#### 2.2.1 Preparation and modification of MnO_2_ nanoparticles

4 mg of potassium permanganate and 8 mg of PAH were both dissolved in 40 ml of ultrapure water and the mixture was stirred for 10 min. Next, the mixed solution was transferred into Ultra-15 Centrifugal Filter Units (MWCO: 100 kDa) under 4000 rpm for 10 min to purify MnO_2_ nanoparticles. The purification process was repeated with ultrapure water for three times. After that, MnO_2_ nanoparticles were obtained by centrifugation at 12500 rpm for 5 min to remove any large particles. For PEGylation, MnO_2_ aqueous solution (10 mg) was mixed with PAA aqueous solution (10 mg) with magnetic stirring at room temperature for 4 h. Next, the above solution was transferred into Ultra-15 Centrifugal Filter Units (MWCO: 100 kDa) under 4000 rpm for 5 min to remove unbound PAA. After ultra-centrifugation, the solution was reacted with mPEG-NH_2_ (50 mg) and EDC (15 mg) under magnetic stirring at room temperature overnight, followed by the ultra-centrifugation to obtain the MnO_2_@PEG nanoparticles.

#### 2.2.2 Preparation of MnO_2_@CaCO_3_ nanoparticles

MnO_2_@CaCO_3_ nanoparticles were synthesized by gas diffusion method. Briefly, 250 μl of MnO_2_ aqueous solution was added into 100 ml of anhydrous ethanol containing 40 mg of CaCl_2_ under sonication in a glass beaker. The glass beaker was put into a sealed chamber containing 3 g of NH_4_HCO_3_. After maintaining the whole system at 40°C for 10 h, MnO_2_@CaCO_3_ nanoparticles were obtained by centrifugation at 10,000 rpm for 15 min and re-dispersed in ethanol for further drug loading and modification.

#### 2.2.3 Preparation and modification of MnO_2_@CaCO_3_@AS nanoparticles

2.5 ml of MnO_2_@CaCO_3_ solutions (4 mg/ml) was added with 0.5 ml of AS ethanol solution (10 mg/ml) under magnetic stirring at room temperature for 2 h. The MnO_2_@CaCO_3_@AS nanoparticles were obtained by centrifugation at 10,000 rpm for 15 min and re-dispersed in ethanol. For the surface modification, the MnO_2_@CaCO_3_@AS nanoparticles then were mixed with 1 ml of DOPA chloroform solution (2 mg/ml) under sonication for 20 min. After centrifugation to remove excess DOPA, the precipitation was re-suspended with 2 ml of chloroform, following by the addition of 1 ml of DPPC chloroform solution (4 mg/ml), 1 ml of cholesterol chloroform solution (2 mg/ml) and 1 ml of DSPE-PEG_2k_ chloroform solution (8 mg/ml). The mixture was stirred overnight at room temperature. Finally, the MnO_2_@CaCO_3_@AS@PEG nanoparticles were collected by evaporation and re-dispersion in ultrapure water for further use.

### 2.3 Characterization of nanoparticles

#### 2.3.1 Stability of nanoparticles

Nanoparticles without PEGylation (MnO_2_@CaCO_3_@AS) or with PEGylation (MnO_2_@CaCO_3_@AS@PEG) were dispersed into ultrapure water, PBS and RPMI-1640 containing 10% FBS, respectively. The particle size of nanoparticles at each time point was monitored by Nano ZS90 Malvern Zetasizer (Malvern, England).

#### 2.3.2 Drug loading capacity and encapsulation efficiency of artesunate in nanoparticles

The quantification of AS was detected by UV-vis-NIR spectrometer (PerkinElmer, United States). Briefly, nanoparticles were dispersed in 2% NaOH solution containing 50% ethanol and incubated for 30 min at 60°C to allow AS being completely converted into UV-absorbing compounds. After cooling down to room temperature and centrifugation to remove any large particles, the supernatant was used to measure the loading content of AS by recording the absorbance at 290 nm. The DLC% and EE% of AS in nanoparticles were calculated as followed:

$$DLC\;\%=\frac{Weight\;of\;drug\;in\;nanoparticles}{Weight\;of\;nanoparticles}\times 100\;\%$$

$$EE\;\%\;\frac{Weight\;of\;drug\;in\;nanoparticles}{Weight\;of\;initial\;drug\;added}\times 100\;\%$$

*pH-responsive and GSH-responsive release profile of nanoparticles*: To measure the tumor microenvironment-triggered release of manganese ions and AS from nanoparticles, MnO_2_@CaCO_3_@AS@PEG was placed into a dialysis bag (MWCO: 3500) and immersed with 10 ml buffer solution at pH 7.4 (10 mM) and pH 5.6 (10 mM) with or without GSH (10 mM) for different times. At designated time points, 1 ml of solution was collected to determine the release profile and then 1 ml of fresh buffer solution was added. The concentration of manganese ions was determined by inductively coupled plasma mass spectrometry (Thermo Fisher, United States), and the concentration of AS was measured by UV-vis-spectrometry.

### 2.4 Investigation of reactive oxygen species generation and glutathione depletion

#### 2.4.1 Reactive oxygen species generation from Mn^2+^-mediated Fenton-like reaction

MB (10 μg/ml), H_2_O_2_ (1 mM), Mn^2+^ (0.5 mM) and HCO_3_^−^ (6.25, 12.5, 25, 50, or 100 mM) were incubated in 1 ml of aqueous solutions at room temperature for 30 min. The generation of ROS was monitored by the decreasing absorbance at 660 nm.

#### 2.4.2 Reactive oxygen species generation from MnO_2_ nanoparticles

MB (10 μg/ml), H_2_O_2_ (1 mM), MnO_2_ (0.5 mM equivalent to Mn^2+^) and GSH (0.1, 0.5, 1, 5 and 10 mM) were incubated in 1 ml of aqueous solutions at room temperature for 30 min. The generation of ROS was monitored by the decreasing absorbance at 660 nm.

#### 2.4.3 Detection of glutathione depletion

GSH (0.5 mM) and MnO_2_ (0.1, 0.2, 0.4, 0.6, 0.8, and 1.0 mM) were incubated in 990 μL aqueous solution at room temperature for 10 min. Next. 10 μL of DTNB solutions (10 mM, pH 8.3) was added into the above solutions. After incubation at room temperature for 10 min, the depletion of GSH was monitored by the decreasing absorbance at 412 nm.

#### 2.4.4 Reactive oxygen species generation from artesunate

TMB (1 mg/ml), AS (10 mM) and Mn^2+^ (2.5, 5, 10, 20, 40 and 60 mM) were incubated in 1 ml DMSO-H_2_O solutions (v/v, 4/6) at room temperature for 30 min. The generation of ROS was monitored by the increasing absorbance at 660 nm.

### 2.5 Cellular experiments

Murine CT26 colon cancer cells obtained from American Type Culture Collection (ATCC) were cultured with Roswell Park Memorial Institute (RPMI) 1640 medium containing 10% fetal bovine serum and 1% penicillin/streptomycin in standard condition (37°C, 5% CO_2_).

#### 2.5.1 Investigation of cellular internalization and mechanism

CT26 cells pre-seeded in 24-well cell culture plates (5 x 10^4^ cells per well) were incubated with DiR-labeled MnO_2_@CaCO_3_@AS@PEG nanoparticles for different times (0, 1, 2, and 4 h). After removal of cell culture medium containing nanoparticles, cells were rinsed by PBS and stained with Hoechst 33342 at 37°C for 10 min. Next, cells were washed with PBS and observed by DMi8 fluorescence microscope (Leica, Germany). For quantification analysis, cells were collected and then analyzed with a flow cytometer (Beckman Coulter, America) to monitor the fluorescence signal. To investigate the mechanism of cellular internalization, CT26 cells were pre-treated with chlorpromazine hydrochloride (20 μM), genistein (200 μM), amiloride hydrochloride (100 μM) and colchicine (10 μM) or pre-incubated at 4°C for 1 h, following by the incubation with DiR-labeled MnO_2_@CaCO_3_@AS@PEG nanoparticles for another 2 h. After being stained with Hoechst 33342, cells were observed by fluorescence microscope for visualization and were analyzed by flow cytometry for quantification.

#### 2.5.2 Evaluation of cytotoxic effect of nanoparticles on cancer cells

CT26 cells pre-seeded in 96-well cell culture plates (1 x 10^4^ cells per well) were treated with AS or nanoparticles for 24 h. After removal of the supernatant, cells were added with MTT and incubated at 37°C for 4 h. Next, the MTT solution was removed and 100 μl of DMSO was added into each well of cell culture plate. The cell viability was then measured with microplate reader (Molecular Devices, Canada) by detecting the absorbance at 490 nm.

#### 2.5.3 Live/dead staining assay

CT26 cells pre-seeded in 24-well culture plate (5 x 10^4^ cells per well) were treated with AS, MnO_2_@CaCO_3_@PEG and MnO_2_@CaCO_3_@AS@PEG for 24 h. The concentration of AS, Mn^2+^ and Ca^2+^ were 0.2 mM, 0.2 mM and 1.0 mM, respectively, in the corresponding group. Next, the live/dead staining assay of treated cells was analyzed by Calcein/PI Cell Viability/Cytotoxicity Assay Kit (Beyotime, China).

#### 2.5.4 Colony formation assay

CT26 cells pre-seeded in 6-well culture plate (500 cells per well) was treated with AS, MnO_2_@CaCO_3_@PEG and MnO_2_@CaCO_3_@AS@PEG for 24 h. The concentration of AS, Mn^2+^ and Ca^2+^ were 0.1 mM, 0.1 mM and 0.5 mM, respectively, in the corresponding group. Next, the cells were cultured in fresh medium for 10 days. Finally, CT26 cells were fixed with 4% paraformaldehyde at room temperature for 15 min and subsequently were stained with crystal violet for 15 min.

#### 2.5.5 Cell cycle assay

CT26 cells pre-seeded in 24-well culture plate (5 x 10^4^ cells per well) were treated with AS, MnO_2_@CaCO_3_@PEG and MnO_2_@CaCO_3_@AS@PEG for 24 h. The concentration of AS, Mn^2+^ and Ca^2+^ were 0.1 mM, 0.1 mM and 0.5 mM, respectively, in the corresponding group. Next, the cell cycle assay of treated cells was analyzed by Cell Cycle and Apoptosis Analysis Kit (Beyotime, China).

#### 2.5.6 Detection of intracellular Ca^2+^ content

CT26 cells pre-seeded in 24-well culture plate (5 x 10^4^ cells per well) were treated with AS, MnO_2_@CaCO_3_@PEG and MnO_2_@CaCO_3_@AS@PEG for 12 h. The concentration of AS, Mn^2+^ and Ca^2+^ were 0.2, 0.2, and 1.0 mM, respectively, in the corresponding group. After removal of the supernatant, cells were stained with intracellular calcium ion indicators Fluo-4 AM and Hoechst 33,342 for 20 min. Next, cells were observed by fluorescence microscope for visualization and were analyzed by flow cytometry for quantification.

#### 2.5.7 Mitochondrial potential detection

CT26 cells pre-seeded in 24-well culture plate (5 x 10^4^ cells per well) were treated with AS, MnO_2_@CaCO_3_@PEG and MnO_2_@CaCO_3_@AS@PEG for 24 h. The concentration of AS, Mn^2+^ and Ca^2+^ were 0.2, 0.2, and 1.0 mM, respectively, in the corresponding group. After removal of the supernatant, cells were stained with Rhd 123 and Hoechst 33342 for 20 min. Next, cells were observed by fluorescence microscope for visualization.

#### 2.5.8 Investigation of intracellular glutathione level

CT26 cells pre-seeded in 24-well culture plate (5 x 10^4^ cells per well) were treated with AS, MnO_2_@CaCO_3_@PEG and MnO_2_@CaCO_3_@AS@PEG for 12 h. The concentration of AS, Mn^2+^ and Ca^2+^ were 0.2, 0.2, and 1.0 mM, respectively, in the corresponding group. After removal of the supernatant, cells were stained with ThiolTracker™ Violet for 20 min, following by the observation with fluorescence microscope and quantification with flow cytometry.

#### 2.5.9 Investigation of intracellular reactive oxygen species level

CT26 cells pre-seeded in 24-well culture plate (5 x 10^4^ cells per well) were treated with AS, MnO_2_@CaCO_3_@PEG and MnO_2_@CaCO_3_@AS@PEG for 24 h. The concentration of AS, Mn^2+^ and Ca^2+^ were 0.2, 0.2, and 1.0 mM, respectively, in the corresponding group. Next, cells were stained with DCFH-DA and Hoechst 33342 for 20 min, following by the observation with fluorescence microscope and quantification with flow cytometry.

#### 2.5.10 Investigation of intracellular lipid peroxidation level

CT26 cells pre-seeded in 24-well culture plate (5 x 10^4^ cells per well) were treated with AS, MnO_2_@CaCO_3_@PEG and MnO_2_@CaCO_3_@AS@PEG for 24 h. The concentration of AS, Mn^2+^ and Ca^2+^ were 0.2, 0.2, and 1.0 mM, respectively, in the corresponding group. Next, cells were stained with BODIPY™ 493/503 and Hoechst 33342 for 20 min, following by the observation with fluorescence microscope and quantification with flow cytometry.

### 2.6 Statistical analysis

All data were presented as mean ± SD without specifying. The significant difference was determined by the student’s *t-test* using GraphPad Prism 7. *p*-value less than 0.05 (denoted as *), 0.01 (denoted as **) and 0.001 (denoted as ***) was considered statistical significance.

## 3 Results and discussions

### 3.1 Investigation of reactive oxygen species generation from manganese-based materials

**FIGURE 1:**
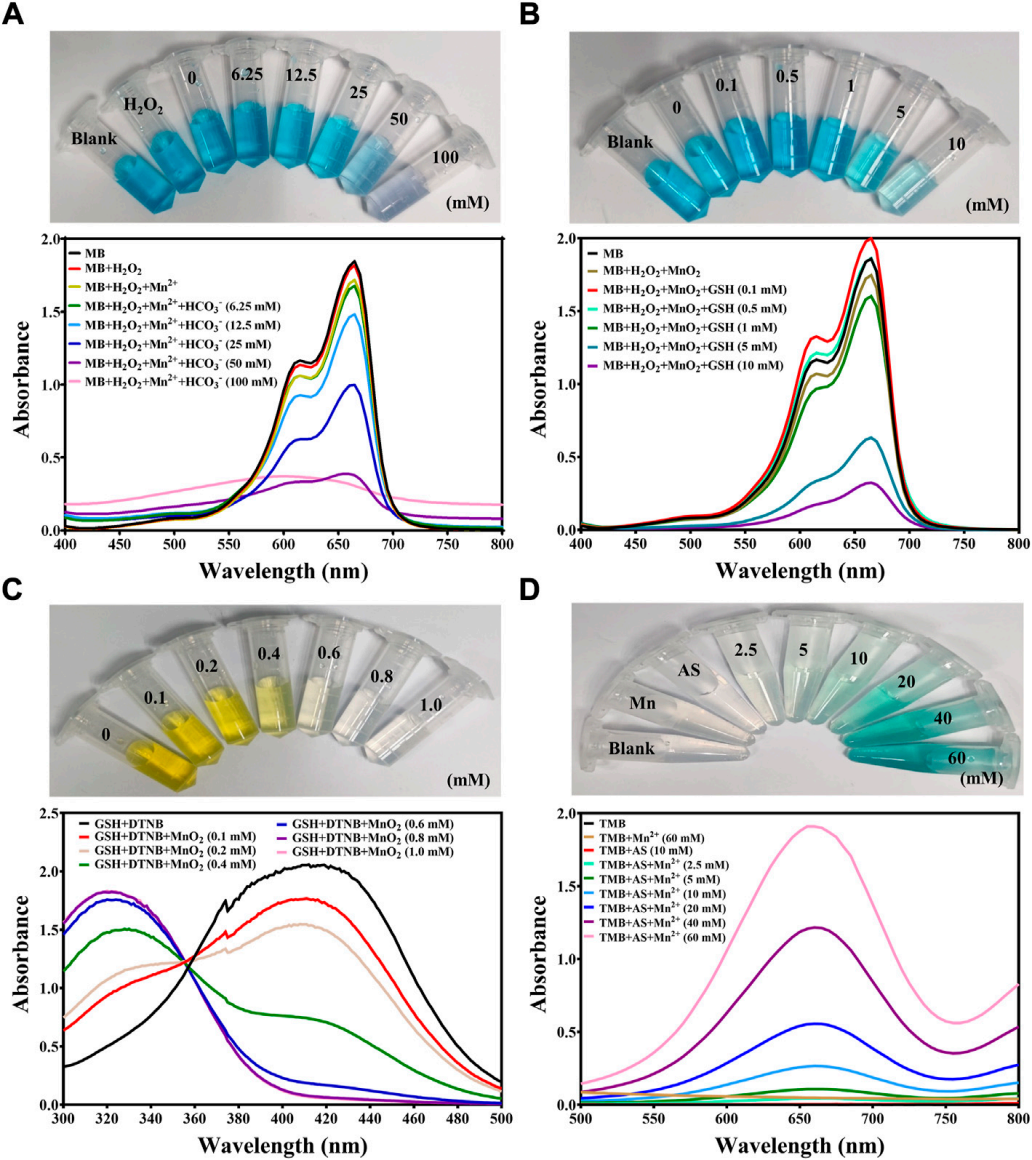
Investigation of ROS generation from manganese-based materials. **(A)** UV-Vis-NIR spectrum of MB (10 μg/ml) before and after being incubated in H_2_O_2_ (1 mM), Mn^2+^ (0.5 mM) and HCO_3_^−^ (6.25, 12.5, 25, 50 or 100 mM) at room temperature for 30 min. The inserted photo represented the color change of MB before and after being incubated in different solutions at room temperature for 30 min **(B)** UV-Vis-NIR spectrum of MB (10 μg/ml) before and after being incubated in H_2_O_2_ (1 mM), MnO_2_ (0.5 mM equivalent to Mn^2+^) and GSH (0.1, 0.5, 1, 5, and 10 mM) at room temperature for 30 min. The inserted photo represented the color change of MB before and after being incubated in different solutions at room temperature for 30 min **(C)** UV-Vis-NIR spectrum exhibiting the GSH depletion of solutions when incubated with GSH (0.5 mM), DTNB (0.1 mM) and MnO_2_ (0.1, 0.2, 0.4, 0.6, 0.8 and 1.0 mM) at room temperature. The inserted photo represented the color change of GSH depletion process. **(D)** UV-Vis-NIR spectrum of TMB (1 mg/ml) before and after being incubated with AS (10 mM) and Mn^2+^ (2.5, 5, 10, 20, 40, and 60 mM) at room temperature for 30 min. The inserted photo represented the color change of TMB before and after being incubated in different solutions at room temperature for 30 min.

We firstly evaluated ROS generation ability of manganese-based materials with different concentrations of HCO_3_^−^. Methylene blue (MB) was used as an indicator of ·OH to reflect the efficiency of Mn^2+^-mediated Fenton-like reaction. As shown in Figure 1A, no obvious decrease in the MB absorbance and color fading was observed when MB was mixed with Mn^2+^ ions and H_2_O_2_ solution, indicating that Mn^2+^ ion-mediated Fenton-like reaction yielded insufficient ROS in the absence of HCO_3_^−^. When the concentration of HCO_3_^−^ gradually increased, an obvious decrease in MB absorbance at 660 nm was monitored by UV-Vis-NIR spectrum and the blue color of MB gradually faded, implying that HCO_3_^−^ was indispensable in Mn^2+^-mediated ROS generation. Also, the degradation level of MB was correlated with the concentration of Mn^2+^ ions (Supplementary Figure S1A) and the reaction time (Supplementary Figure S1B). Collectively, these studies revealed that Mn^2+^-driven Fenton-like reaction can effectively produce ·OH in physiological environment. Furthermore, the ·OH-induced MB degradation was greatly inhibited in the presence of GSH as shown in Supplementary Figure S2, revealing that GSH as an intracellular antioxidant significantly scavenged ROS and impeded the therapeutic effect of CDT. Thus, a manganese-based materials with the ability of GSH depletion was highly desired to achieve efficient CDT.

It has been reported that MnO_2_ could consume GSH to enhance various dynamic therapy (Liu Q. et al., 2022; Guan et al., 2022; Sun et al., 2022). Therefore, we examined the possibility of using MnO_2_ nanoparticles to generate toxic ·OH as well as exhaust intracellular GSH. It was found that MnO_2_ nanoparticles obviously degraded MB in the presence of GSH (Figure 1B, Supplementary Figures S2, S3), which was attributed to the GSH-responsive Mn^2+^ release from MnO_2_ to initiate the ROS generation. In the meanwhile, the MnO_2_ was on-demanded degradation in response to GSH in a concentration-dependent manner (Supplementary Figure S4). Next, the GSH depletion ability of MnO_2_ nanoparticles was determined. As shown in Figure 1C, DTNB was used to detect the residual concentration of GSH to yield a yellow color product with strong absorbance at 412 nm. When MnO_2_ nanoparticles were added, GSH was rapidly oxidized into GSSG, indicating the ability of MnO_2_ to exhaust GSH (Figure 1C).

Delivering exogenous peroxide compounds that generate ROS with the assistance of transition metal ions would avoid the restriction of insufficient endogenous peroxide compounds and boost the ROS generation. AS, a natural product with an endoperoxide bridge, is found to produce ROS in the presence of manganese ions (Wang D. et al., 2021). Hence, we further examined the ROS generation ability of AS in the presence of manganese-based materials. It was discovered that TMB was oxidized into blue-color compound ox-TMB and stronger absorbance at 660 nm was recorded when the concentration of Mn^2+^ ions increased, implying that AS served as a fuel to aggravate the intracellular ROS level with the assistance of manganese-based materials (Figure 1D and Supplementary Figure S5). Collectively, we hypothesized that MnO_2_ nanoparticles could serve ideal igniters to explode the endogenous peroxide pool and artesunate could serve as fuel to replenish the peroxide pools. Therefore, synchronous delivery of MnO_2_ nanoparticles and AS would be a promising strategy to intensify the intracellular oxidative stress for efficient tumor eradication.

### 3.2 Preparation and characterization of MnO_2_@CaCO_3_@AS@PEG nanoparticles

**FIGURE 2:**
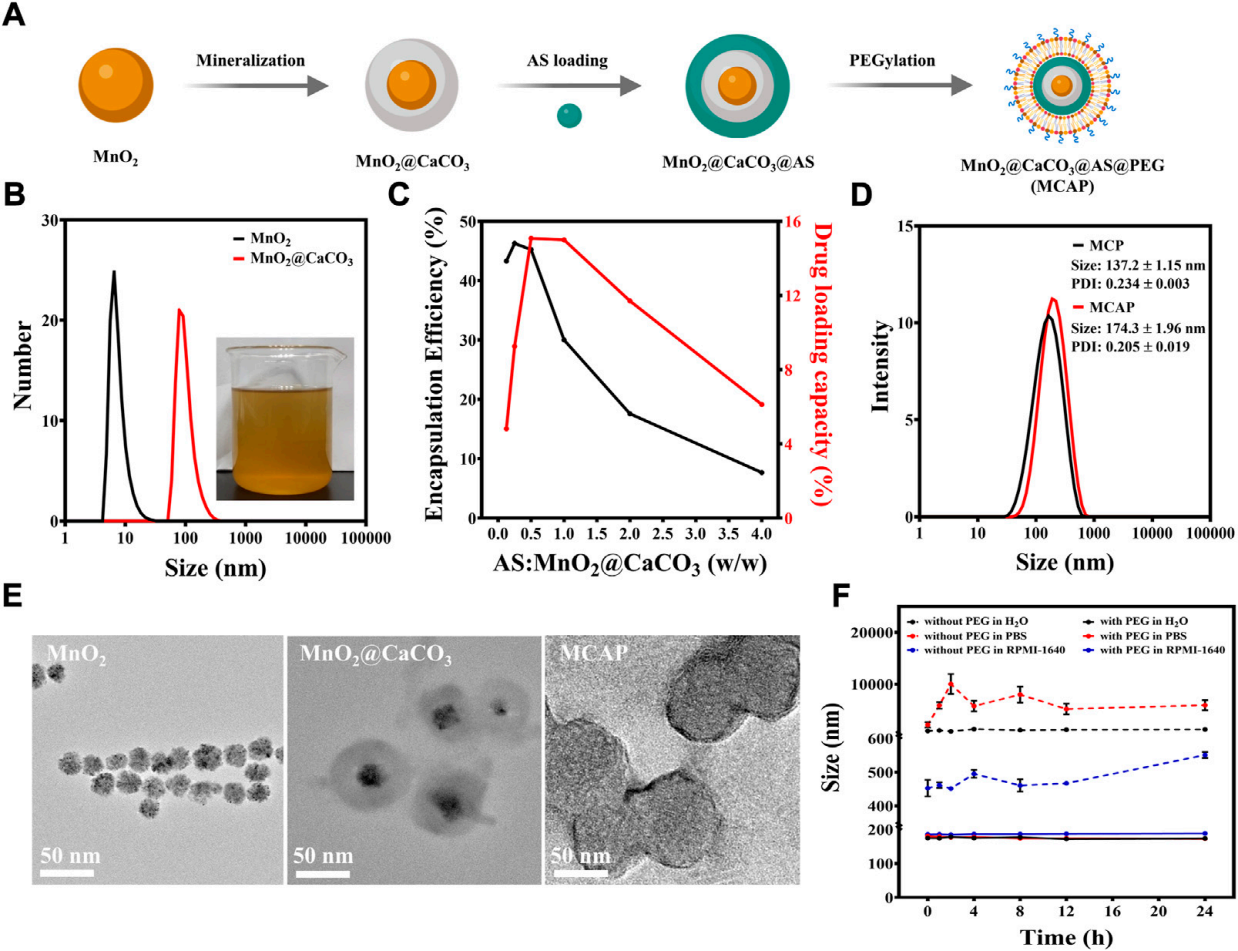
Preparation and characterization of MCAP NPs. **(A)** The preparation procedure of MCAP NPs. **(B)** DLS measurements of MnO_2_ and MnO_2_@CaCO_3_ nanoparticles in ethanol. The inserted photo represents the MnO_2_@CaCO_3_ nanoparticles in ethanol synthesized through the gas-diffusion method. **(C)** Quantification of AS loading capacity of MnO_2_@CaCO_3_ nanoparticles at different AS:MnO_2_@CaCO_3_ feeding ratios. **(D)** DLS measurement of MnO_2_@CaCO_3_@PEG (MCP) nanoparticles and MnO_2_@CaCO_3_@AS@PEG (MCAP) nanoparticles in aqueous solutions. **(E)** Respective TEM images of MnO_2_ nanoparticles, MnO_2_@CaCO_3_ nanoparticles and MCAP NPs. **(F)** Time-dependent DLS variations of MnO_2_@CaCO_3_@AS nanoparticles with or without PEGylation incubated in different solutions for 24 h.

The preparation procedure of MnO_2_@CaCO_3_@AS@PEG nanoparticles (denoted as MCAP NPs) was illustrated in Figure 2A to achieve co-delivery of MnO_2_ and AS. Briefly, MnO_2_ nanoparticles were firstly prepared through permanganates reduction method (Supplementary Figure S6). The transmission electron microscopy (TEM) images showed that the obtained MnO_2_ was a spherical structure with the average size of about 10 nm (Figure 2E). To encapsulate AS, MnO_2_ nanoparticles were subsequently coated with CaCO_3_ *via* a one-pot gas diffusion method. The core-shell structure of MnO_2_@CaCO_3_ revealed that MnO_2_ was successful coated by a layer of CaCO_3_ (Figure 2E). The as-prepared MnO_2_@CaCO_3_ nanoparticle showed dark yellow color and its hydrodynamic size was increased to about 100 nm (Figure 2B). Due to the coordination interaction between Ca^2+^ ion and carboxyl group of AS, the CaCO_3_ shell coated on MnO_2_ nanoparticles served as anchor points for AS loading. As displayed in Supplementary Figure S7B, the peak at 730 cm^−1^ and 825 cm^−1^ were observed in MnO_2_@CaCO_3_@AS nanoparticles, which was assigned to the endoperoxide bridge (O-O) of AS (Vacque et al., 1997; Wan et al., 2019; Chen et al., 2021). Besides, the C=O stretching vibrations of AS showed a characteristic peak at 1758 cm^−1^ in Supplementary Figure S7C. When AS was loaded on the CaCO_3_-based nanoparticles, the peak was observed to shift about 10 cm^−1^ to 1748 cm^−1^, implying that calcium ions coordinated with the carboxyl group of AS (Byler and Farrell, 1989). By simply mixing AS and MnO_2_@CaCO_3_ nanoparticles in ethanol at a mass feeding ratio of 0.5:1 at room temperature for 2 h, AS was self-assembly on the surface of MnO_2_@CaCO_3_ and the drug-loading nanoparticles (MnO_2_@CaCO_3_@AS) with relative high AS encapsulation efficiency (∼45.2%) and high AS loading capacity (∼15.1%) were obtained. (Supplementary Figure S8 and Figure 2C).

Then, MnO_2_@CaCO_3_@AS nanoparticles were modified with a lipid bilayer through previous methods to confer better physiological stability for tumor-specific ROS amplification (Zhong et al., 2022). The dynamic light scattering (DLS) measurement exhibited that the hydrodynamic size of MnO_2_@CaCO_3_@AS@PEG nanoparticles (MCAP NPs) was ∼174 nm. In contrast, the hydrodynamic size of MnO_2_@CaCO_3_@PEG nanoparticles was ∼137 nm, which was slightly smaller than that of their corresponding AS-loaded counterparts (Figure 2D). To investigate whether modification of nanoparticles with lipid bilayer could improve their physiological stability, MnO_2_@CaCO_3_@AS nanoparticles with or without PEGylation were incubated with different solutions including ultrapure water, PBS and cell culture medium for 24 h. It was found that MnO_2_@CaCO_3_@AS nanoparticles with PEGylation exhibited negligible fluctuation in all testing conditions during the incubation time. Additionally, the hydrodynamic size and polydispersity index of MnO_2_@CaCO_3_@AS nanoparticles with PEGylation were less than 200 nm (Figure 2F) and 0.3 (Supplementary Figure S9), respectively. However, the hydrodynamic size of MnO_2_@CaCO_3_@AS nanoparticles without PEGylation showed huge variation in testing conditions and large aggregates were observed in PBS solutions (Figure 2F), implying that surface modification with lipid bilayers indeed endow the nanoparticles with excellent stability in a physiological environment.

Given that the concentration of intracellular GSH within cancer cells is higher than that in normal cells and the endo-/lysosomal lumen maintains low pH, MCAP NPs were then incubated in normal physiological environment (pH 7.4) as well as cancer intracellular condition (pH 5.6 + GSH) to evaluate the drug release profile. It was found that less Mn^2+^ and AS were released from MCAP NPs in a normal physiological environment, which was ascribed to the inert property of CaCO_3_ and MnO_2_ in a neutral solution. However, more than 80% of Mn^2+^ and AS were released from MCAP NPs in mimic intracellular conditions (Supplementary Figure S10). This could be ascribed to the pH-responsive degradation of CaCO_3_ and MnO_2_ as well as GSH-sensitive disassembly of MnO_2_, together accelerating the decomposition of MCAP NPs and the release of therapeutic cargoes for tumor-specific ROS amplification therapy.

### 3.3 Cellular uptake and mechanism

**FIGURE 3:**
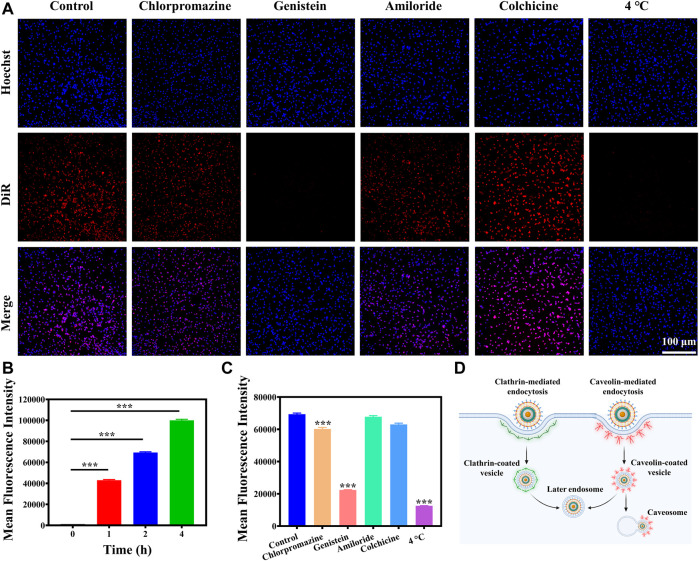
Cellular uptake and mechanism. **(A)** Fluorescence images of CT26 cells pre-treated with inhibitors and then incubated with DiR-labelled MCAP NPs for 2 h. **(B)** Flow cytometry analysis of CT26 cells incubated with DiR-labelled MCAP NPs for different times. **(C)** Flow cytometry analysis of CT26 cells pre-treated with inhibitors and then incubated with DiR-labelled MCAP NPs for 2 h **(D)** A scheme illustrated the possible cellular uptake mechanism and intracellular trafficking of MCAP NPs in CT26 cells (***p < 0.001, **p < 0.01, *p < 0.05).

Efficient cellular uptake of nanoparticles is essential for cancer cell eradication. Therefore, it is necessary to investigate the endocytosis pathways of MCAP NPs firstly. MCAP NPs were labeled with red fluorescent probes DiR to visualize the cellular internalization process. As shown in Supplementary Figures S11A,B and Figure 3B, CT26 cells incubated with DiR-labelled MCAP NPs exhibited a time-dependent internalization profile. A strong fluorescence signal was observed when CT26 cells were incubated with nanoparticles for 4 h, demonstrating the efficient internalization of MCAP NPs by CT26 cells. It is the prerequisite to achieving efficient tumor eradication by aggravating the intracellular oxidative stress using ROS nanoamplifiers MCAP NPs.

Next, we further investigated the internalization mechanism of nanoparticles by pre-treating cells with inhibitors or in 4°C environment. Almost no red fluorescence signal was detected when CT26 cells were pre-incubated in 4°C condition, manifesting that CT26 cells internalized MCAP NPs in an energy-dependent mechanism (Figures 3A,C and Supplementary Figure S11C). Then, CT26 cells were pre-treated with different uptake inhibitors for 1 h including chlorpromazine (clathrin-mediated endocytosis inhibitor), genistein (caveolae-mediated endocytosis inhibitors), amiloride (micropinocytosis inhibitors) and colchicine (microtubules associated transport inhibitors). Fluorescence imaging and flow cytometry analysis revealed that CT26 cells pretreated with chlorpromazine and genistein exhibited lower intracellular uptake of MCAP NPs as compared to control groups and other inhibitors pre-treatment groups (Figures 3A,C and Supplementary Figure S11C), displaying that MCAP NPs entered CT26 cells *via* clathrin-/caveolae-mediated endocytosis. Collectively, we proposed the possible cellular uptake mechanism and intracellular trafficking of MCAP NPs based on the experiment results and related literature. As illustrated in Figure 3D, the ROS nanoamplifiers MCAP NPs were internalized by CT26 colon cancer cells through clathrin-/caveolin-mediated endocytosis and this endocytosis process relied on an energy-dependent mechanism. On one hand, MCAP NPs entrapped in the clathrin-coated vesicle would fuse with early endosomes to form late endosomes, which eventually fused with lysosomes (Kaksonen and Roux, 2018). The lower pH environment of lysosomes as well as the higher GSH content within cancer cells would trigger the burst release of Mn^2+^ ions and AS (Supplementary Figure S10) to intensify the intracellular ROS level. On the other hand, MCAP NPs entrapped in caveolin-coated vesicles would partially fuse with endosomes or form caveosomes to deliver cargo to the Golgi apparatus and endoplasmic reticulum (Donahue et al., 2019).

### 3.4 *In vitro* anticancer effect of MnO_2_@CaCO_3_@AS@PEG nanoparticles on CT26 cells

**FIGURE 4:**
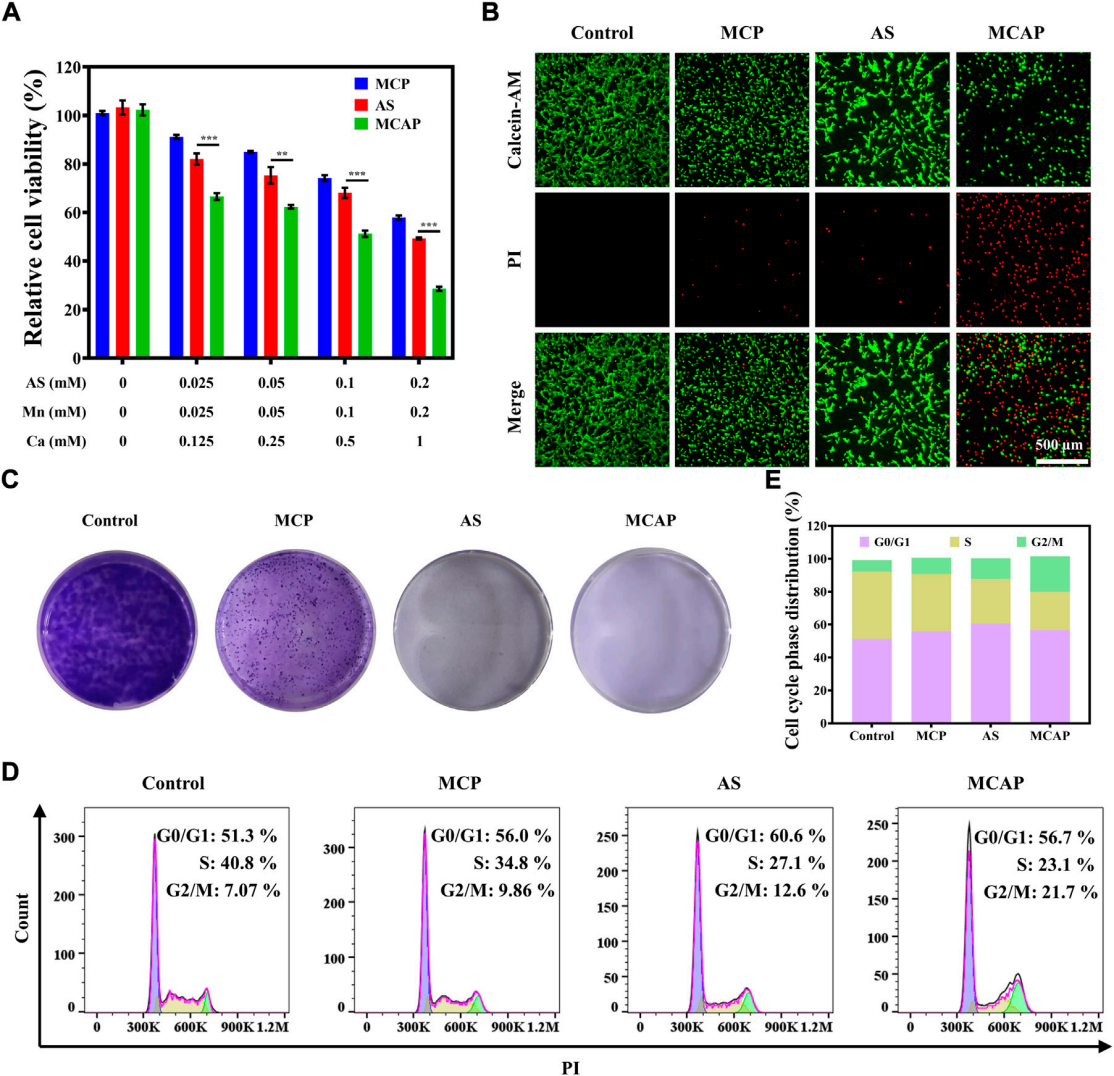
*In vitro* anticancer effect of MCAP NPs on CT26 cells. **(A)** Relative cell viability of CT26 cells after being treated with AS, MCP NPs and MCAP NPs for 24 h. **(B)** Fluorescence images of CT26 cells treated with various formulations and stained with Calcein-AM (live cells: green color) and PI (dead cells: red color). **(C)** The colony formation of CT26 cells after various treatments. **(D)**. **(E)** Flow cytometry analysis of cell cycle phase distribution of CT26 cells after various treatments (***p < 0.001, **p < 0.01, *p < 0.05).

After careful investigation of cellular uptake profile of CT26 cells on MCAP NPs, we then evaluated the cytotoxic effect of nanoparticles on cellular level. As illustrated in Figure 4A, AS exhibited moderate inhibition effect on CT26 cells. Additionally, MCP NPs that were composed of MnO_2_ and CaCO_3_ also displayed moderate cytotoxicity on CT26 cells. Among various treatment approaches, MCAP NPs that synchronously delivered MnO_2_, AS and CaCO_3_ showed the highest cytotoxic effect on CT26 cancer cells, which was consistent with the results of live-dead staining (Figure 4B). Afterward, we analyzed how the compositions of MCAP NPs demonstrated the synergistic anticancer effect on CT26 cells. As shown in Supplementary Figure S12, physically mixing AS and MnO_2_ exhibited a stronger inhibition effect than AS or MnO_2_ along, which indicated that AS and MnO_2_ might enhance the anticancer efficiency of each other in a synergistic mechanism. Similarly, physically combining AS and CaCO_3_ also showed an enhanced cytotoxic effect on CT26 cells as compared to single treatment at low concentrations. Taken together, the highest cytotoxic effect of MCAP NPs among various treatment approaches might be related to the synergistic effect among AS, MnO_2_ and CaCO_3_.

The colony formation assay is a widely accepted method to assess the reproductive viability of cancer cells after being exposed to various treatment strategies. As shown in Figure 4C, CT26 cells treated with various agents exhibited impaired ability to form colonies compared with control group. Most importantly, cells being exposed to AS and MCAP NPs displayed the least capacity in colony formation, which may provide hints for the potential application of ROS nanoamplifiers MCAP NPs in *in vivo* anticancer evaluation. Finally, we estimated the cell cycle phase distribution of CT26 cells after treatments. As compared to the control group, treatments with MCP NPs, AS and MCAP NPs induced different levels of G2/M phase arrest in CT26 cells. Among all, CT26 cells treated with MCAP NPs exhibited the longest extension at the G2/M phase, indicating that intracellular DNA in this treatment group might suffer from severe damage (Figures 4D,E) (Wang et al., 2016). This observation was consistent with other ROS-mediated anticancer therapies as accumulated ROS within cancer cells were reported to induce G2/M phase arrest (Gong et al., 2020; Kong et al., 2020; Tan et al., 2020). Overall, CT26 cells treated with ROS nanoamplifiers MCAP NPs displayed the highest cytotoxicity and increased arrest at G2/M phase, contributing to the superior suppression of colony formation.

### 3.5 Intracellular oxidative stress amplification of MnO_2_@CaCO_3_@AS@PEG nanoparticles

Inspired by the efficient cancer eradication effect of MCAP NPs on CT26 cells, the intracellular oxidative stress caused by ROS nanoamplifiers MCAP NPs were further evaluated. Considering that CaCO_3_ was one of the components in the nanoparticles, and calcium-based nanomaterials have been utilized to amplify tumor oxidative stress through Ca^2+^-overloading-mediated mitochondrial dysfunction, we firstly determined the intracellular Ca^2+^ ion level as well as mitochondrial membrane potential. As illustrated in Supplementary Figure S13, CT26 cells treated with AS, MCP NPs and MCAP NPs all displayed different levels of elevated intracellular Ca^2+^ ion content. Interestingly, AS was found to upregulate the intracellular concentration of Ca^2+^ ions, which might be related to the inhibition of sarcoplasmic/endoplasmic reticulum Ca^2+^-ATPase (Fernández-Martínez et al., 2013; Wan et al., 2019). This finding was consistent with previous studies (Nagamune et al., 2007; Zhou et al., 2013; Wang et al., 2019), revealing the potential of AS in combination with calcium-based nanomaterials for Ca^2+^-overload cancer therapy as well as providing the preliminary explanation for the synergistic anticancer effect of AS and CaCO_3_ (Supplementary Figure S12). CT26 cells treated with MCAP NPs exhibited the highest intracellular Ca^2+^ ion content among various treatment groups, which was ascribed to the introduction of exogenous Ca^2+^ ion by CaCO_3_ and upregulation of intracellular Ca^2+^ ion by AS. It is reported that Ca^2+^-overload would be contributed to cancer cell death through mitochondrial dysfunction (Kerkhofs et al., 2018) and oxidative stress (Peng and Jou, 2010). Therefore, we evaluated the mitochondrial membrane potential of CT26 cells after being treated with various formulations by using commercial Rhd 123 dye as indicators. As shown in Supplementary Figure S14, CT26 cells without any treatment exhibited the strongest fluorescence signal, indicating the normal mitochondrial membrane potential. However, cells treated with AS, MCP NPs and MCAP NPs displayed different levels of impaired membrane potential, as indicated by the decreased fluorescence signal. Importantly, almost no green fluorescence signal was detected in cells treated with ROS nanoamplifiers MCAP NPs. Collectively, ROS nanoamplifiers MCAP NPs could effectively induce Ca^2+^ ion overloading and cause severe depolarized membrane potential.

**FIGURE 5:**
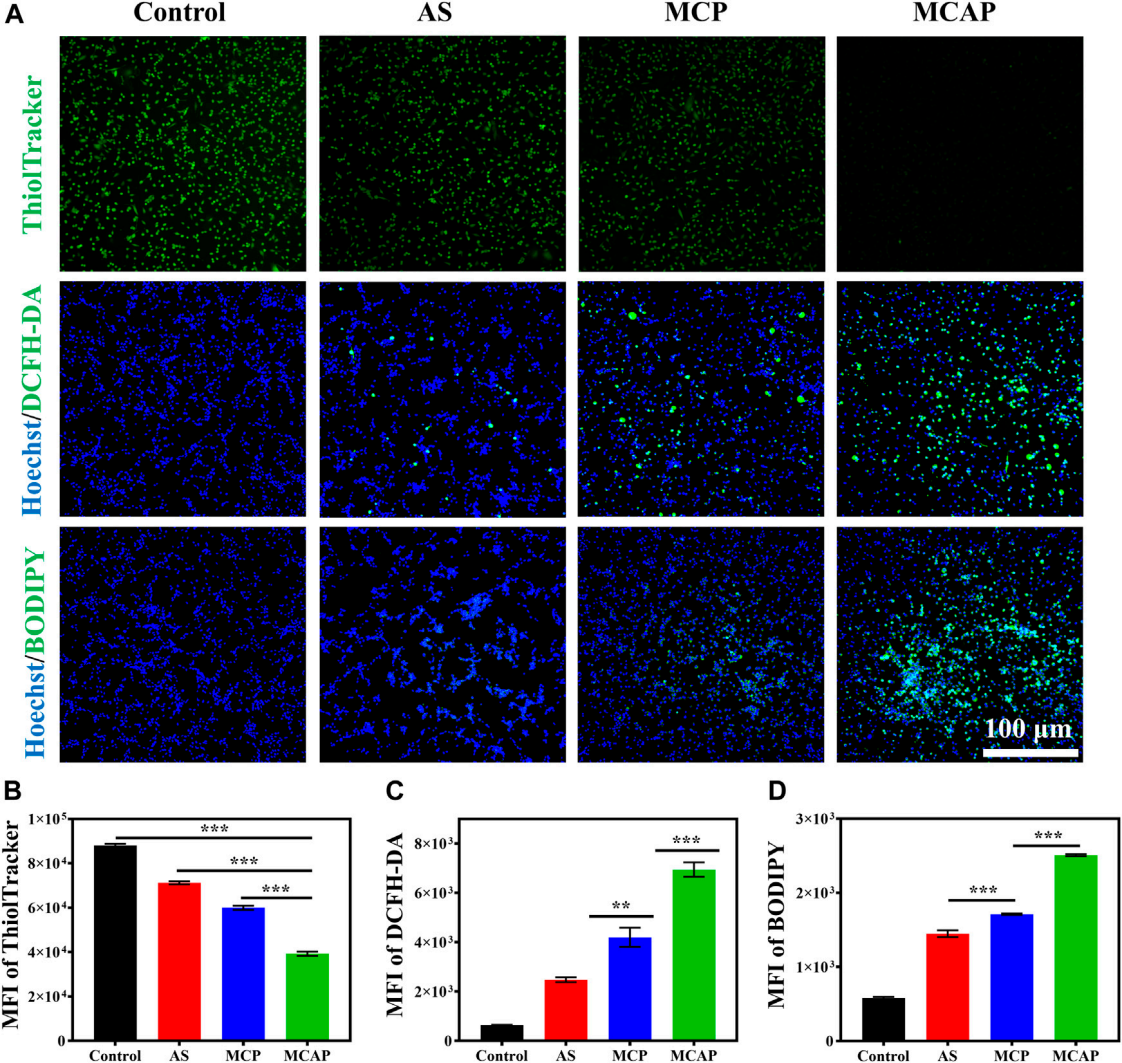
Intracellular oxidative stress amplification of MCAP NPs. **(A)** Fluorescence images of CT26 cells treated with various agents and then stained with ThiolTracker™ Violet (GSH: green color), Hoechst 33342 (nuclei: blue color), DCFH-DA (ROS: green color) and BODIPY (lipid peroxidation: green color). Flow cytometry analysis of CT26 cells treated with various agents then stained with **(B)** ThiolTracker™, **(C)** DCFH-DA and **(D)** BODIPY (***p < 0.001, **p < 0.01, *p < 0.05).

Eventually, we investigated the intracellular oxidative stress amplification of CT26 cells treated with AS and nanoparticles. It has been reported that MnO_2_ possessed the ability to exhaust intracellular GSH (Lin L.-S. et al., 2018; Liu Q. et al., 2022). Therefore, we studied the effect of various MnO_2_-based nanomaterials on GSH depletion using a commercial intracellular GSH probe ThiolTracker™ Violet dye. As observed under the fluorescence microscope, the CT26 colon cancer cells generated the strongest green fluorescence signal (Figure 5A), which may be attributed to the high concentration of intracellular GSH. In contrast, cells treated with AS displayed reduced green fluorescence signal, indicating that AS could partially consume intracellular GSH. Besides, cells treated with MCP NPs showed lower green fluorescence signal (Figure 5B) due to the catalytic reduction from Mn^4+^ ions to Mn^2+^ ions accompanied by GSH exhaustion. Obviously, cells internalized with MCAP NPs that simultaneously delivered MnO_2_ and AS demonstrated the lowest green fluorescence signal, revealing that the intracellular GSH was rapidly exhausted. The robust depletion of GSH thus resulted in the disturbance of cellular antioxidant defense system, creating a beneficial environment for oxidative stress damage by nanoamplifiers. Subsequently, we analyzed the intracellular ROS levels of CT26 cells treated with various agents. Fluorescence imaging and flow cytometry revealed that cells treated with AS showed mild ROS generation, which was attributed to the catalytic cleavage of endoperoxide bridge of AS by free ferric ions within cancer cells (Figures 5A,C and Supplementary Figure S15). Additionally, MCP NPs treatment induced moderate ROS generation on CT26 cells. Moreover, cells receiving MCAP NPs treatment exhibited the highest intracellular ROS level, which was resulted from the multiple amplification of tumor oxidative stress including Mn^2+^-mediated Fenton reaction (Figures 1A,B), GSH depletion (Figures 1C, 5A), AS-boosted CDT (Figure 1D) and Ca^2+^-overloading-mediated mitochondrial dysregulation (Supplementary Figure S14). The generated ROS then attacked intracellular macromolecules, such as polyunsaturated fatty acids of plasma membrane, to induce cancer cell death. As shown in Figures 5A,D and Supplementary Figure S16, both AS and MCP NPs treatment induced moderate lipid peroxidation levels within CT26 cells, as indicated by the green fluorescence signal from BODIPY probe. However, cells treated with MCAP NPs displayed the highest level of lipid peroxidation, majorly attributing to the highest ROS production and most efficiently GSH exhaustion from the nanoamplifiers MCAP NPs. Taken together, ROS nanoamplifiers MCAP NPs could significantly aggravate intracellular oxidative stress to achieve efficient tumor eradication.

**SCHEME 1:**
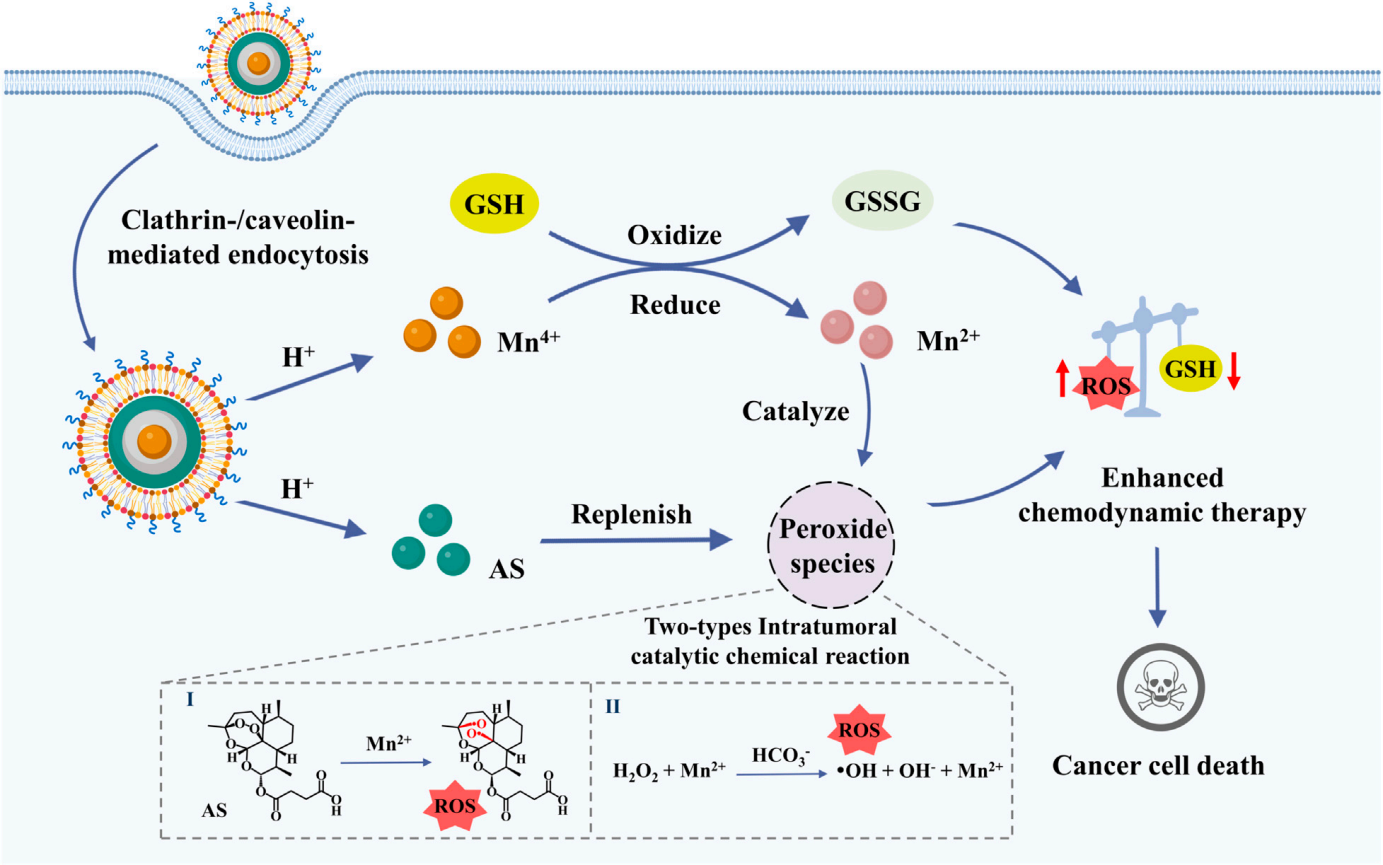
Efficient tumor eradication was achieved *via* multiple amplification of intratumoral oxidative stress through GSH exhaustion and Mn^2+^-mediated intratumoral catalytic reaction of H_2_O_2_ and AS.

## 4 Conclusion

In this study, we have prepared an oxidative stress nanoamplifier with ROS amplification and GSH exhaustion (MCAP NPs) *via* layer-by-layer assembly of AS and CaCO_3_ coating on the surface of MnO_2_. In tumor acidic and reduced microenvironment, the nanoamplifier was disassembled to release AS and Mn^2+^ to achieve amplification of intratumoral oxidative stress *via* multiple pathways. Concretely, the released AS served as exogenous peroxide compounds to replenish intratumoral peroxide species, facilitating the Mn^2+^-catalyzed ROS production. Furthermore, MnO_2_ consumed GSH to yield Mn^2+^ for ROS generation through intratumoral catalyzation of H_2_O_2_ and AS, jointly disturbing redox balance and eventually enhancing the CDT efficacy. In conclusion, this work presents a facile and promising oxidative stress nanoparticle with ROS amplification and GSH exhaustion to enhance CDT efficacy in cancer treatment.

## Data Availability

The raw data supporting the conclusion of this article will be made available by the authors, without undue reservation.
