# Supplementary material for: A multifunctional oxidative stress nanoamplifier with ROS amplification and GSH exhaustion for enhanced chemodynamic therapy
Source: Front Pharmacol. 2022 Nov 10;13:1044083. doi: 10.3389/fphar.2022.1044083 (PMC9689698; doi:10.3389/fphar.2022.1044083)
Supplement: Supplementary file 1 [file DataSheet1.pdf]

Supplementary Material

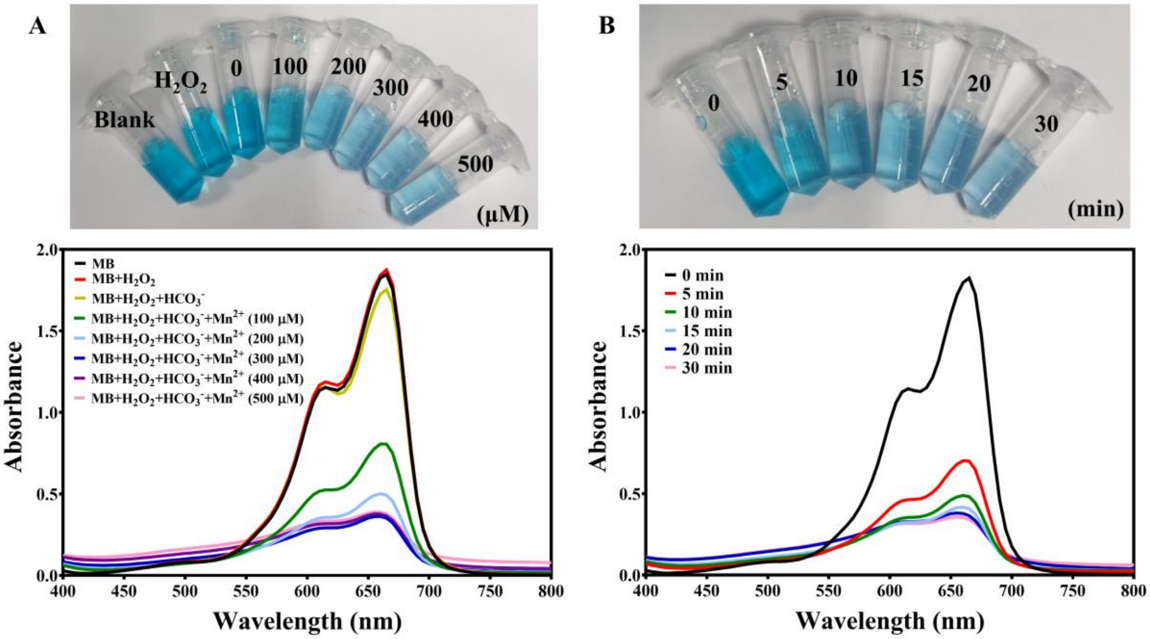

Supplementary Figure S1. Concentration-dependent (A) and time-dependent (B) of Mn^{2+}-mediated Fenton reaction.

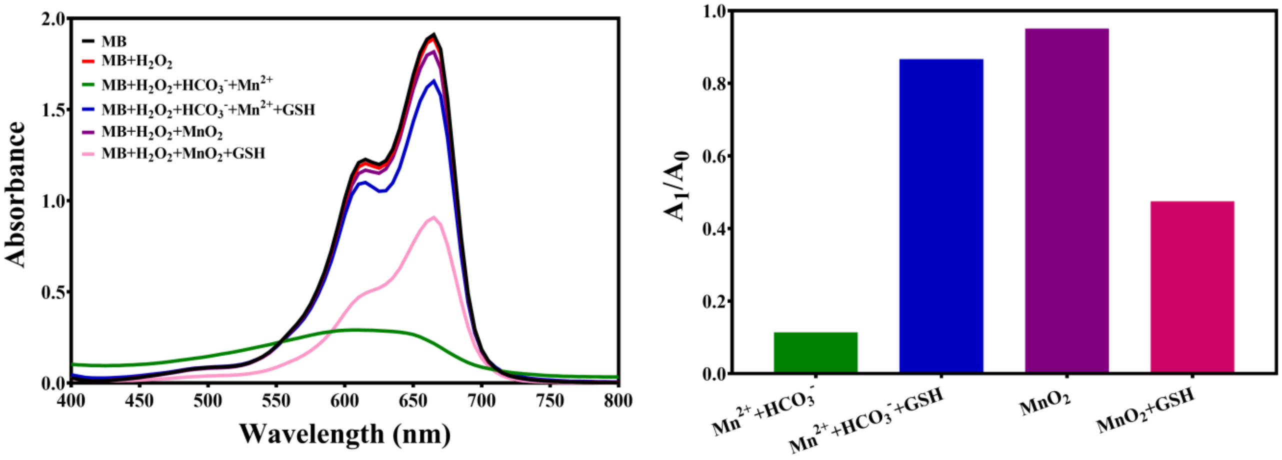

Supplementary Figure S2. Comparison of the Fenton reaction between Mn^{2+} and MnO_2 in different conditions.

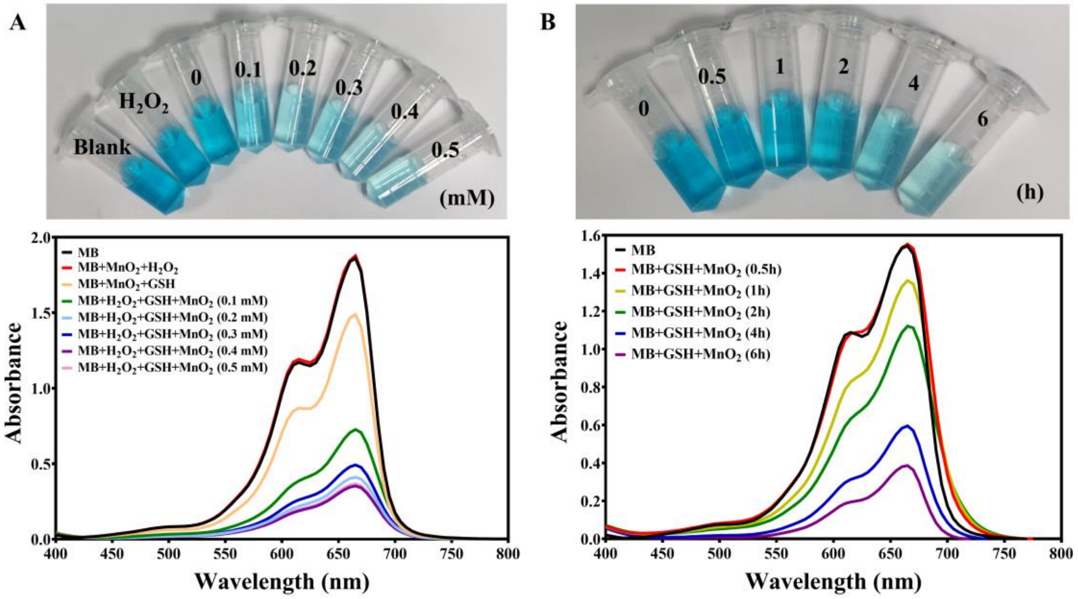

Supplementary Figure S3. Concentration-dependent (A) and time-dependent (B) of MnO_2-mediated Fenton reaction.

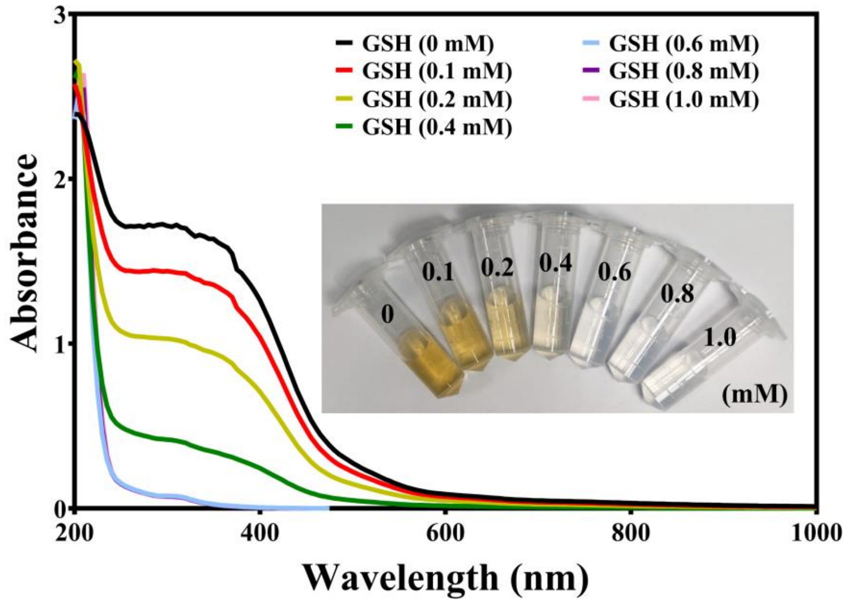

Supplementary Figure S4. Degradation of MnO_2 nanoparticles in different concentrations of GSH.

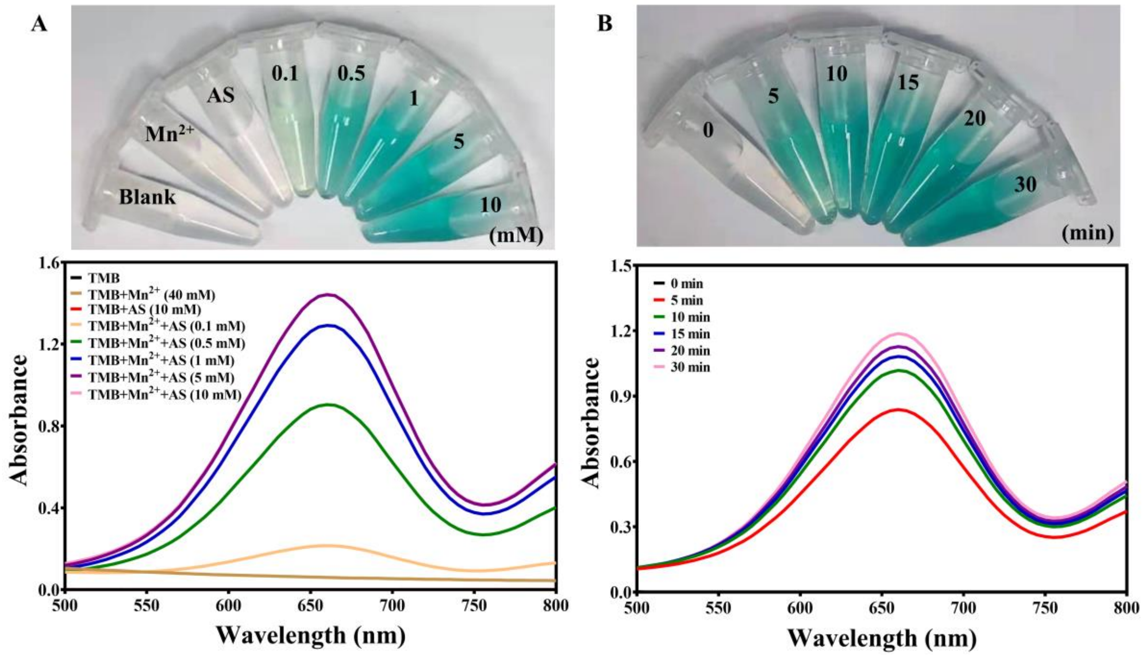

Supplementary Figure S5. Concentration-dependent (A) and time-dependent (B) of AS-mediated Fenton reaction.

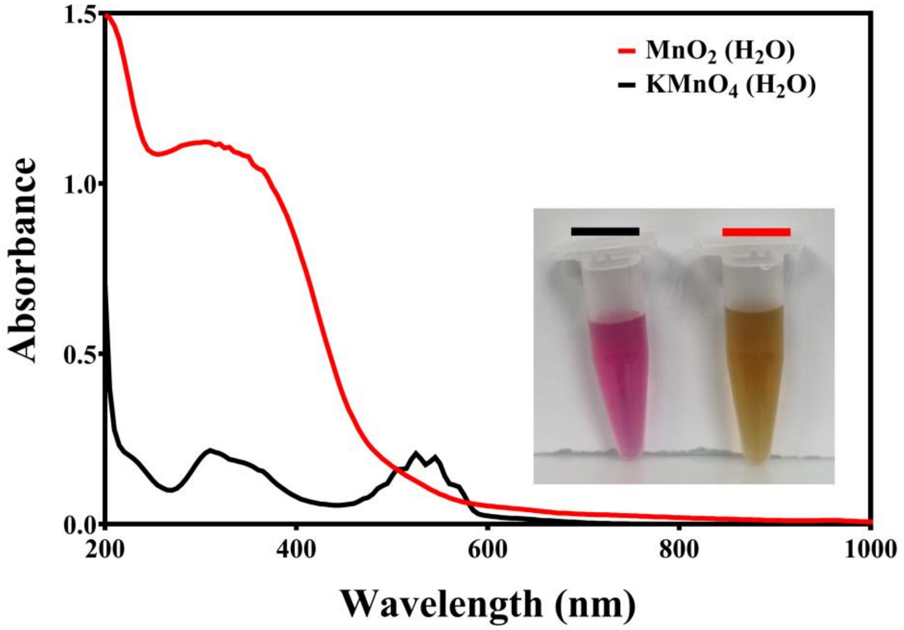

Supplementary Figure S6. UV-Vis-NIR spectrum of MnO_2 nanoparticles solution and $KMnO_4$ solution.

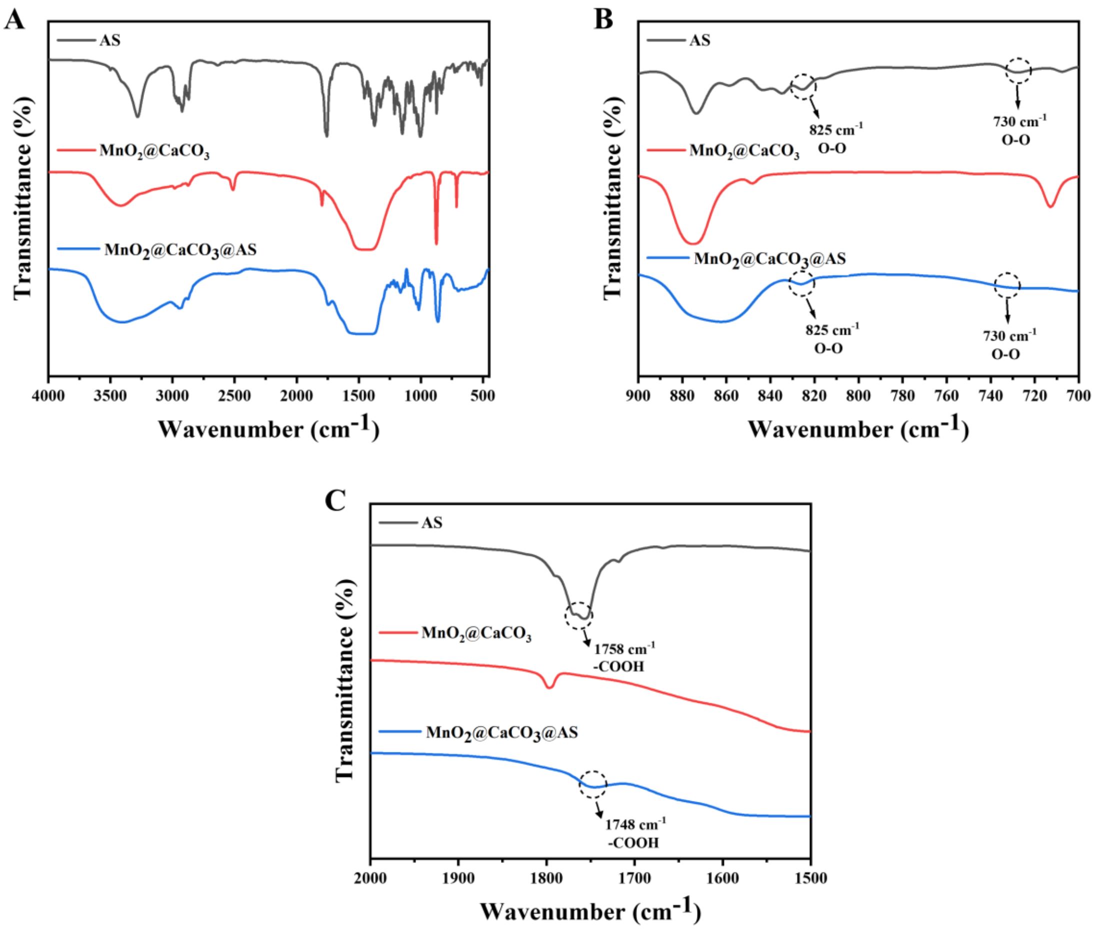

Supplementary Figure S7. FT-IR spectrum of AS, MnO_2@$CaCO_3$ and MnO_2@$CaCO_3$@AS in the wavenumber between (A) 450 – 4000 cm^{-1}; (B) 700 – 900 cm^{-1}; (C) 1500 – 2000 cm^{-1}.

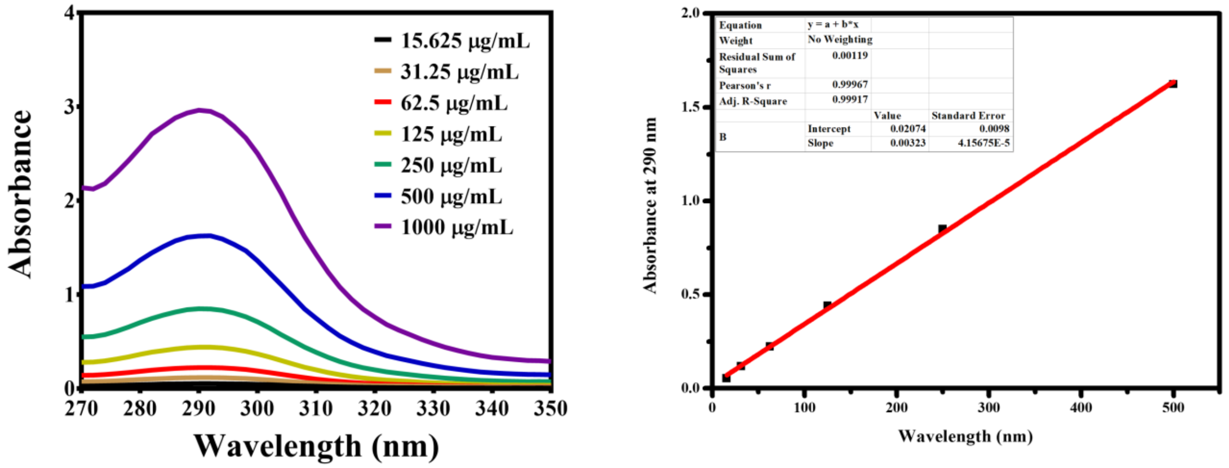

Supplementary Figure S8. Establishment of the standard curve of AS for quantification.

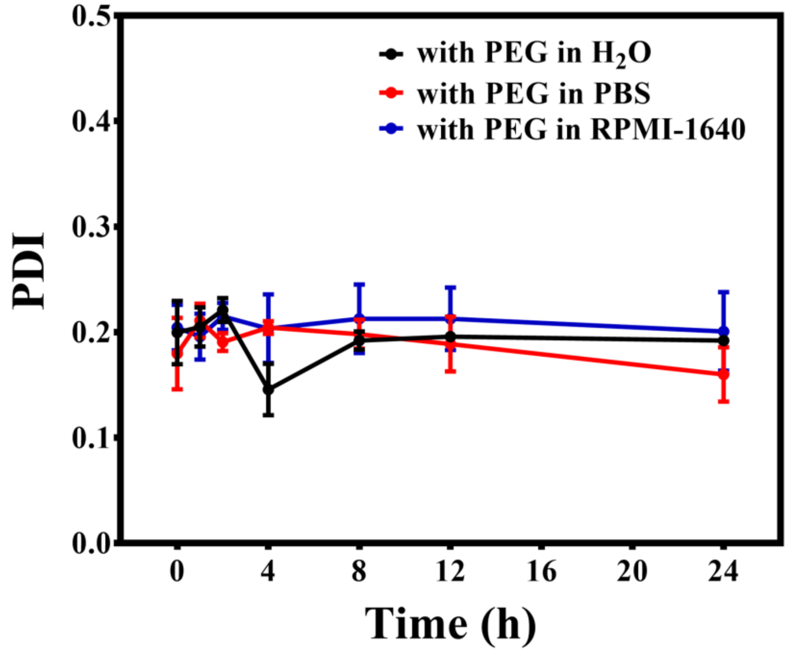

Supplementary Figure S9. PDI of MCAP nanoparticles in different solutions.

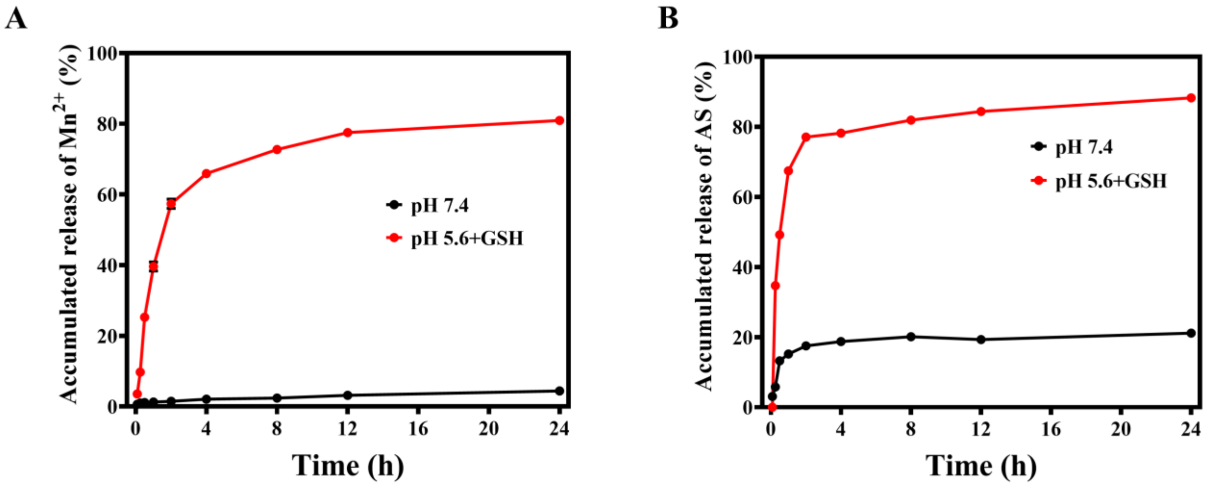

Supplementary Figure S10. Time-dependent release profile of (A) Mn^{2+} and (B) AS from MCAP NPs incubated in different conditions.

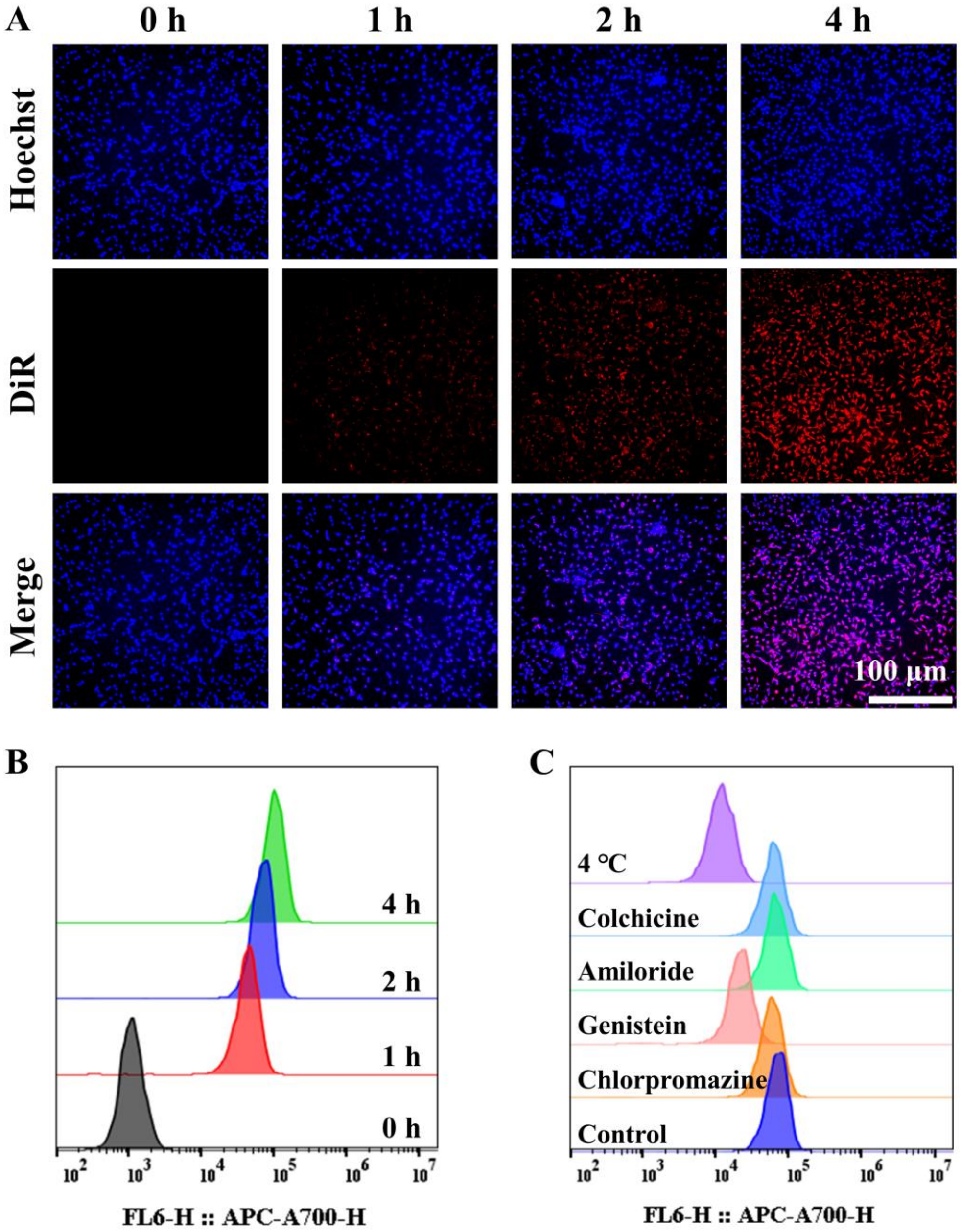

Supplementary Figure S11. (A) Fluorescence images of CT26 cells incubated with DiR-labelled MCAP NPs (red color) for different times and stained with Hoechst 33342 (nuclei: blue color). Flow cytometry analysis of cellular uptake without (B) or with (C) inhibitors.

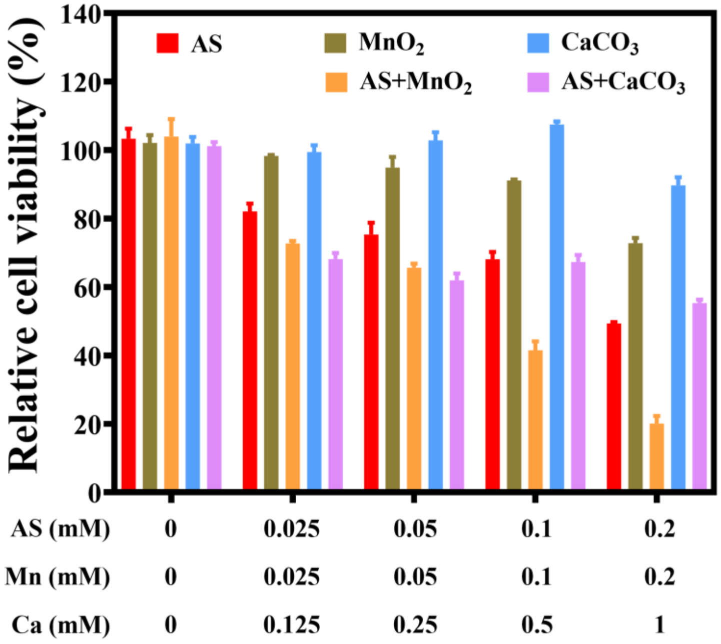

Supplementary Figure S12. Relative cell viability of CT26 cells after being treated with different formulations for 24h.

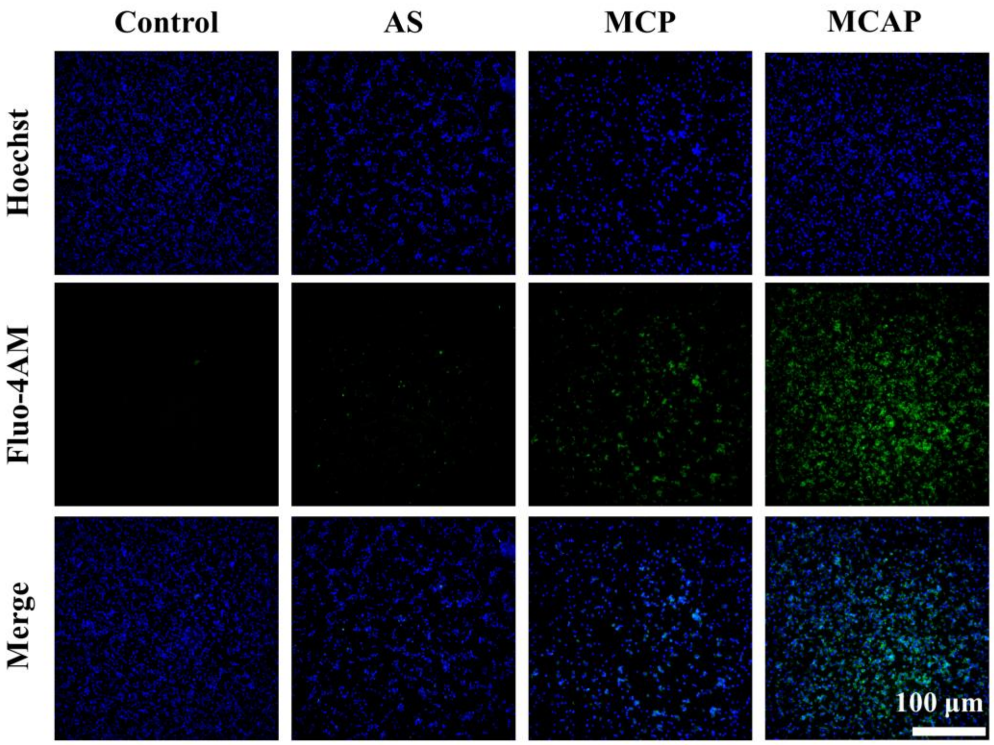

Supplementary Figure S13. Fluorescence images of CT26 cells treated with different formulations for 12 h and then stained with Hoechst 33342 (nuclei: blue color) and Fluo-4AM (intracellular Ca^{2+}: green color).

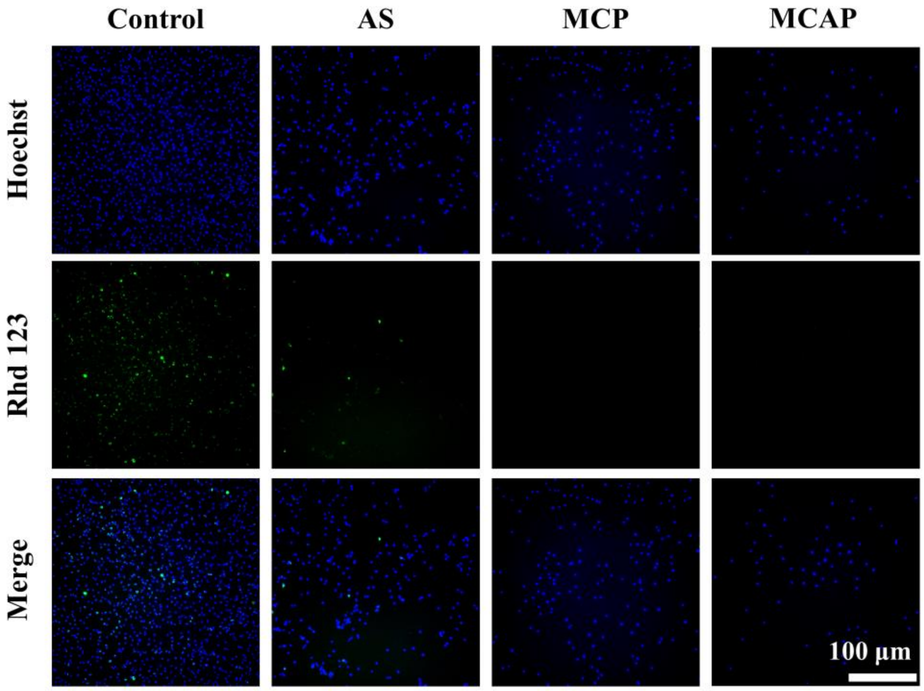

Supplementary Figure S14. Fluorescence images of CT26 cells treated with different formulations for 12 h and then stained with Hoechst 33342 (nuclei: blue color) and Rhd 123 (mitochondrial membrane potential: green color).

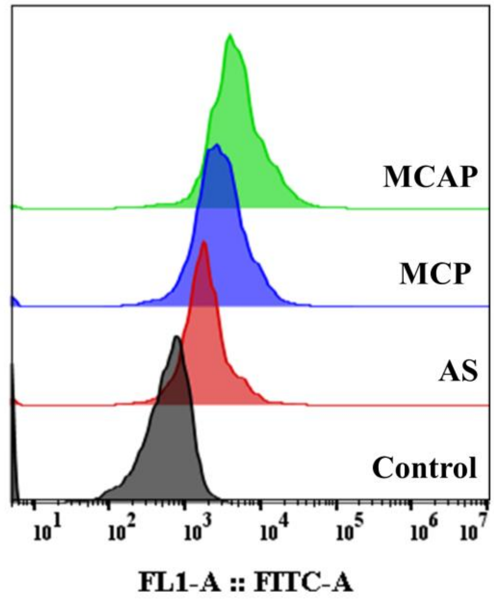

Supplementary Figure S15. Flow cytometer analysis of intracellular ROS after CT26 cells treated with different formulations for 24 h.

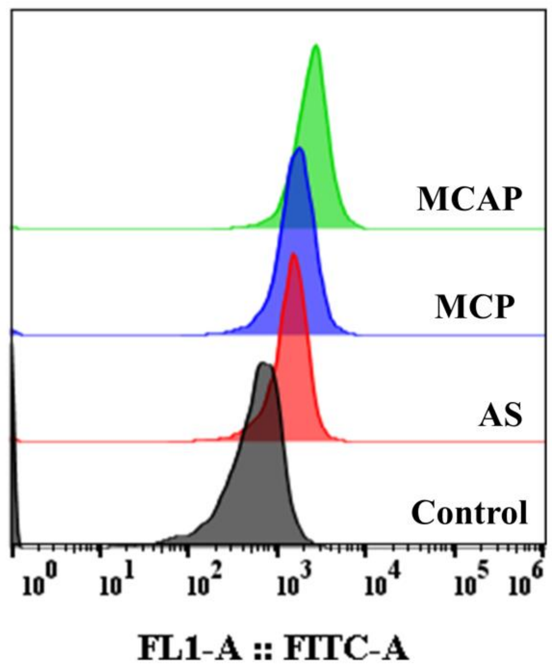

Supplementary Figure S16. Flow cytometer analysis of intracellular lipid peroxidation after CT26 cells treated with different formulations for 24 h.
